# Super Infection of Cutaneous Leishmaniasis Caused by *Leishmania major* and *L. tropica* to *Crithidia fasciculata* in Shiraz, Iran

**Published:** 2019-12

**Authors:** Aliyar MIRZAPOUR, Alireza BADIRZADEH, Marzieh ASHRAFMANSOURI, Hamed BEHNIAFAR, Maryam NOROUZI, Hakim AZIZI, Mahmoodreza BEHRAVAN, Seyyed Javad SEYYED TABAEI

**Affiliations:** 1. Department of Medical Parasitology and Mycology, School of Medicine, Mashhad Branch, Islamic Azad University, Mashhad, Iran; 2. Department of Parasitology and Mycology, School of Medicine, Iran University of Medical Sciences, Tehran, Iran; 3. Department of Medical Parasitology and Mycology, School of Medicine, Shahid Beheshti University of Medical Sciences, Tehran, Iran; 4. Department of Medical Parasitology, Zabol University of Medical Sciences, Zabol, Iran

**Keywords:** ITS1, *Leishmania tropica*, *Leishmania major*, *Crithidia fasciculata*, Iran

## Abstract

**Background:**

The aims of the current study were to determined present status of CL in Shiraz City, identify the causative species of *Leishmania* and conduct phylogenetic evaluations in detected parasites

**Methods:**

This study was conducted on 70 individuals with suspected CL that referred to the major health centers of Shiraz (Valfajr), Fars province, Iran, from Sep 2016 to Jul 2017. DNA was extracted from cultured *Leishmania* promastigotes and PCR-RFLP were performed using ITS1-rDNA gene.

**Results:**

Overall, 39 male (55.70%) and 31 (44.30%) female were found to be positive microscopically. All of direct examined positive samples were confirmed to be positive for *Leishmania* spp. DNA. Based upon the PCRRFLP patterns and phylogenetic analysis, 46 (65.72%), 17 (24.28%) and 7 (10%) isolates were clearly identified as *L. major*, *L. tropica* and *C. fasciculata*, respectively.

**Conclusion:**

The dominat detected species in Shiraz City was *L. major* and *L. tropica*, respectively. CL has high prevalence in Shiraz City; therefore, more studies on leishmaniasis in the natural vectors and also reservoirs infection in this region is exceedingly recommended. Skin leisons due to *C. fasciculata*, was described for the first time in Iran (Shiraz City).

## Introduction

Leishmaniasis (L) is endemic in 98 countries and is considered as major public health problems over the world (1, 2). There are various forms of leishmaniasis, cutaneous (CL), visceral (VL) and mucocutaneous (MCL), caused by the members of the genus *Leishmania* (L) (2). According to WHO, CL is the most common form of the disease with approximately 0.6–1 million new cases in the world (1). Based on causative agents, there are two major forms of CL with several differences in clinical symptoms in Iran, including anthroponotic CL (ACL) and zoonotic CL (ZCL) (1).

Although microscopic examination has been considered the major standard method for identification of CL, due to morphological similarity between different *Leishmania* spp. diagnosis of the main species is not possible (3). Furthermore, identification of causative species is necessary for successful control of CL. Therefore, molecular methods such as restriction fragment length polymorphism PCR (PCR-RFLP) are being used for species identification. Moreover, molecular methods are helpful to evaluate inter-species and intra-species genetic diversity of the genus (4).

The aims of the current study were to determine the present status of CL in Shiraz city, diagnosis of the causative species of disease by using the molecular technique (PCR-RFLP) via ITS1-rDNA gene and conduct phylogenetic evaluations in detected parasites.

## Materials and Methods

### Study area

**Fig. 1:**
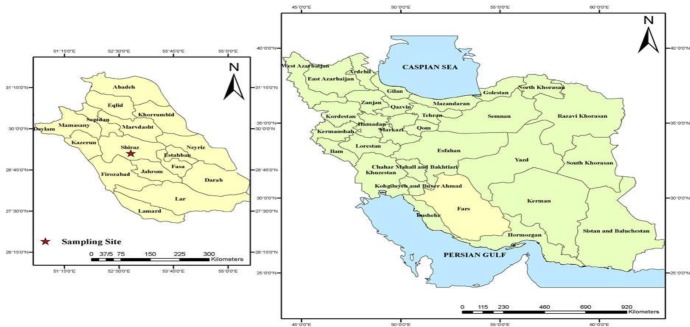
Map of Iran indicating the location of sampling site (Shiraz city) in Fars province, Southwest of Iran (Created by Arc GIS version 10.2)

Current study is a descriptive study conducted from Sep 2016 to Jul 2017 in Shiraz City (Fig. 1). It is one of the southern cities of Iran, located at approximately 1500 meters above sea level. The weather in this city cold and rainy in winter and moderate to hot dry in summer. The annual mean temperature of the city is 18 °C.

### Sampling and parasite culture

Samples were taken from 70 individuals with suspected CL that referred to the major health centers of Shiraz (Valfajr).

Most of the individuals were from urban areas of Shiraz City. After obtaining written informed consent for each participants, demographic information and site of lesion on the body were recorded using a questionnaire (Table 1).

**Table 1:** The characteristics of confirmed CL patients from Shiraz City (Fars Province, Iran) base on personal information and lesion characteristics

***Age Groups (yr)***	***Sex***			***Lesion duration (month)***			***Lesion location***			

	***Total***	***Male***	***Female***	***≤1***	***1–2***	***3≤***	***Head, face and neck***	***Hand and arm***	***Feet and leg***	Other^*^
<9	9	5	4	7	1	2	5	14	2	3
10–19	14	8	6	5	0	8	13	13	11	0
20–29	12	6	6	5	3	4	15	7	2	3
30–39	18	10	8	8	5	5	7	28	8	0
40–49	9	6	3	5	2	2	9	13	7	0
50≤	8	4	4	2	1	4	4	9	2	1
Total	70	39	31	32	12	23	53	70	30	4

Clinical samples (smears) were taken from the swollen margin of the skin lesions of each patient suspected to have CL by sterile vaccine style or lancet. Giemsa stained slides were prepared and examined by light microscopy. Meanwhile at the same time, samples from each CL lesions were aseptically inoculated and cultured into Novy–McNeal–Nicolle (NNN) medium and immediately transported to department of parasitology, Shahid Beheshti University of Medical Sciences, Tehran, Iran.

Informed consent was taken from all patients before taking smear samples. The local Ethics Committee approved the study.

### Genomic DNA extraction

Genomic DNA of isolated parasites from confirmed CL patients was extracted from the stationary growth phase of cultured *Leishmania* promastigotes using the Phenol-chloroform protocol as described elsewhere (5).

### PCR–RFLP for ITS1-rDNA amplification

**Fig. 2:**
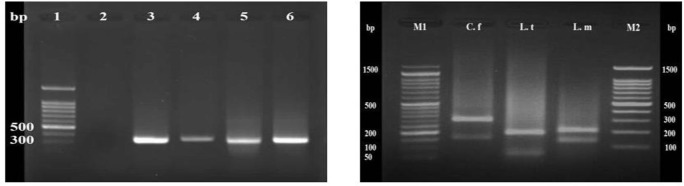
(Left) PCR results of ITS1-rDNA for identification of *Leishmania* sp. Lane 1, 100 bp ladder marker; Lane 2, negative control; Lane 3 and 4, reference strains of *Leishmania* parasites including: *L. tropica* (MHOM/IR/02/M39-Khorasan; Accession Number: JN860725) and *L. major* (MHOM/IR/75/ER; Accession Number: EF653269), respectively; Lanes 5 and 6, positive samples of CL patients due to *Leishmania* infection. (Right) PCR-RFLP analysis of ITS1-rDNA of *Leishmania* spp. using the *HaeIII* restriction enzyme. M1 and M2, 50 bp and 100 bp ladder markers, respectively; C. f, *C. fasciculata* (fragments 350 bp and 150 bp), L.t, *L. tropica* (fragments 57 bp, 56 bp and 200 bp) and L.m, *L. major* (fragments 155 bp and 206 bp) are isolated *Leishmania* spp. from patients

The standard PCR was conducted to identify the *Leishmania* infection by amplifying ribosomal internal transcribed spacer (ITS1) using previously designed specific primers, LITSR (5`- CTGGATCATTTTCCGATG-3`) as Forward primer and L5.8S (5`TGATACCACTTATCGCACTT-3`) as reverse primer (6). Reference strains of *L. tropica* (MHOM/IR/02/M39-Khorasan; Accession Number: JN860725) and *L. major* (MHOM/IR/75/ER; Accession Number: EF653269) were used as positive standard controls (Fig. 2). Next, RFLP was performed on PCR products to determine the parasite species (Fig. 2).

### Sequencing and phylogenetic analyses

Out of the 70 confirmed *Leishmania* isolates, 15 were randomly selected and purified from the agarose gel by PCR purification kit (Bioneer, South Korea) for sequencing analysis (*C. fasciculata* (seven isolates), *L. major* (four isolates) and *L. tropica* (four isolates)). Hence, the first PCR amplicons were sequenced from both directions (forward and reverse) by targeting the ITS1-rDNA gene, same primers in the PCR, according to the Sanger instructions (Bioneer, South Korea). The sequences alignment was done using BLAST program of GenBank and was compared with those of existing sequences related to *Leishmania* spp. which was available in the GenBank database. Eight nucleotide sequences are deposited in the GenBank database under the following accession numbers: MG755819, MG755820 and MG755821 for *L. major*, MG755822, MG755823 and MG755824 for *L. tropica* and MG755817 and MG755818 for *C. fasciculata*.

**Fig. 3:**
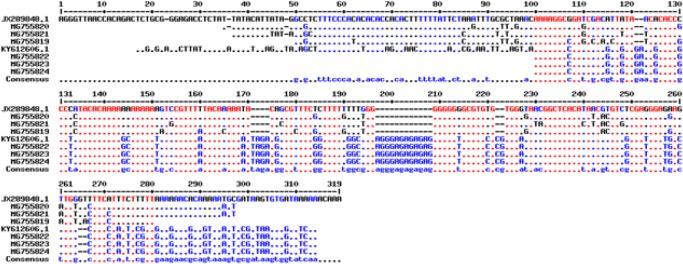
The multiple sequence alignment of ITS1-rDNA gene using online Multalin software (http://multalin.toulouse.inra.fr/multalin/) for comparison of *L. major* and *L. tropica*. Six sequenced *Leishmania* ITS1-rDNA genes in the current study are MG755819, MG755820 MG755821, MG755822, MG755823 and MG755824. *L. tropica* (JX289848.1) isolate MHOM/IR/12/Bam4 and *L. major* isolate MLM-IR88 were used for comparison of similarity and differences between *Leishmania* spp.

**Fig. 4:**
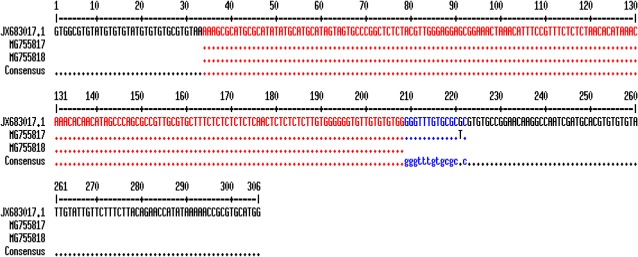
The multiple sequence alignment of ITS1-rDNA gene using online Multalin software (http://multalin.toulouse.inra.fr/multalin/) for comparison of *C. fasciculata*. Two sequenced *Crithidia* ITS1-rDNA genes in the current study are MG755817 and MG755818. *C. fasciculata* (JX683017.1) isolate MHOM/IR/90/M42 was used for comparison of similarity and differences between *Crithidia* spp.

**Fig. 5:**
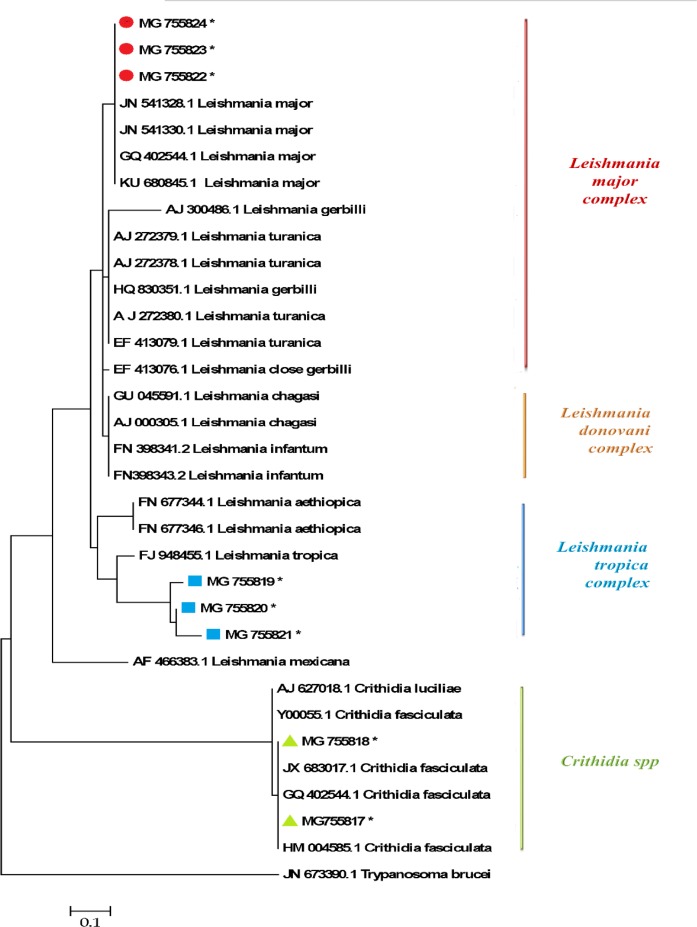
A maximum likelihood phylogenetic tree of *Leishmania* and *Crithidia* spp. based on ITS1-rDNA gene compared with other trypanosomatidae family. Evolutionary analyses were conducted in MEGA 5.05. *L. mexicana* (MNYC/BZ/62/M379; Accession number: AF466383) and *Trypanosoma brucei* (TS07112; Accession number: JN673390) were utilized as out-groups. Sequenced *Leishmania* and *Crithidia* ITS1-rDNA genes in the current study are illustrated in colored symbols with stars

After extraction of the related genes forms Gen-Bank, the multiple sequence alignment was performed using online Multalin software (http://multalin.toulouse.inra.fr/multalin/) (Fig. 3 and 4). In this regard, overlapped sequences (contigs) were edited at each consensus positions by using Sequencher Tmv.4.1.4 Software (Gene Codes Corporation). For demonstrating the precise taxonomic status of all isolates, a maximum likelihood phylogenetic tree was constructed by using the MEGA 5.05 software based on kimura two-parameter model (Fig. 5).

## Results

Seventy patients with confirmed CL lesions, who lived in the CL endemic regions of Shiraz city, were recruited for the current study (Fig. 1). CL diagnosis from each isolates was confirmed by demonstration of parasite amastigotes in the skin smears, culture of isolated promastigotes in RPMI-1640 medium and PCR (Fig. 2). All of the 70 suspected CL patients were found to be positive using microscopic examination and PCR. Species of isolated parasites were identified using PCR-RFLP. Of this, PCR products of ITS1-rDNA gene were digested using *BsuR*I (*Hae*III) restriction enzyme. Therefore, of 70 confirmed CL lesions, 46 (65.72%) and 17 (24.28%) were identified as *L. major* (fragments 155 bp and 206 bp) and *L. tropica* (fragments 57 bp, 56 bp, and 200 bp) respectively, and the rest (seven isolates; 10%) were *C. fasciculata* (fragments 350 bp and 150 bp) (Fig. 2). Notably, no mix-infection was found in identified isolates.

Out of the 70 confirmed CL patients recruited in this study, 30 (44.29%) were female and 39 (55.71%) were male, with the age ranges of 1–65 yr. Most of the patients were belong to 30–39 and 10–19 yr’ age group. Total of 70 lesions were observes on the hands and arms; 53 on the head, face and neck; 30 on the feet and leg; and 4 on the back, belly and waist. Most of the lesions were seen on the hands (Table 1). The lesions duration ranged from 15 d to eight months. None of the patients have undergone any CL treatment prior sampling. Complete description of patients and lesions characteristics including age, sex, number of lesions and duration are illustrated in Table 1.

The multiple sequence alignment using ITS1-rDNA gene for *L .major*, *L. tropica* and *C. fasciculata* is illustrated in Fig. 3 and 4. Of 15 randomly sequenced ITS1-rDNA genes of *Leishmania* and *Crithidia*, eight sequences were chosen and directly compared with extracted GenBank sequences of three *Leishmania* and *Crithidia* reference species including *L. tropica* (JX289848.1) isolate MHOM/IR/12/Bam4, *L. major* isolate MLMIR88 and *C. fasciculata* isolate MHOM/IR/90/M42 Fig. 3 and 4. Totally 33 ITS1-rDNA gene sequences of *Leishmania* and *Crithidia* spp. were prepared for analyzing in order to create the phylogenetic tree including eight sequenced isolates in the current study which registered currently in the GenBank, 18 and five sequences belonging to distinct *Leishmania* complexes and *Crithidia* spp.

Respectively, and two sequences were used as outgroups (*L. mexicana* and *Trypanosoma brucei*). The constructed maximum likelihood phylogenetic tree elucidated that the sequenced *Leishmania* ITS1-rDNA genes in the current study were distributed into two min complexes including *L. major* and *L. tropica*; and *C. fasciculata* distributed into own genus, *Crithidia* (Fig. 5). Interestingly, three sequenced *L. major* from Shiraz City were categorized into the first group close to Iran sequences, and two isolated *C. fasciculata* were grouped in the Iran sequences (Fig. 5). The topology of constructed maximum likelihood phylogenetic tree showed that the *L. tropica* and *L. major* were grouped with the bootstrap value of higher than 60% in their specific complex. The phylogenetic relationship among 31 *Leishmania* and *Crithidia* parasites and two outgroup species is presented in Fig. 5.

## Discussion

This study is the first documented report in the Shiraz city, southern Iran done to identify *Leishmanis* species including *L. major*, *L. tropica* and *C. fasciculata* by molecular methods and confirm them by using sequencing and phylogenetic analysis in clinical samples of CL patients in this city, as one of the main endemic areas in Iran.

Our findings by using ITS-based PCR-RFLP showed that 70 confirmed CL lesions, 46 and 17 were identified as *Leishmania* spp. including *L. major* and *L. tropica* respectively, and seven isolates belonged to *Crithidia* genus, *C. fasciculata*. Moreover, the findings of phylogenetic analysis based upon ITS1-rDNA gene sequences plainly showed close relationship of distinct *Leishmania* and *Crithidia* species, in the constructed maximum likelihood phylogenetic tree.

The dominant species in Shiraz City was *L. major*. However, the results are in agreement with those of other studies in Shiraz Province, in that, the major causative agent of CL in three villages of rural regions of Shiraz is *L. major* (7). Furthermore, another molecular study was done in Fasa City (southeast of Shiraz) which confirmed that *L. major* was predominant species of *Leishmania* in CL patients. Our results about the predominance of *L. major* is in accordance with some of previous studies conducted in different parts of Iran (8). Moreover, unlike rural region of Shiraz, *L. tropica* is the predominant species in urban region of the city (9). The main effectors which cause active transmission in endemic areas can be the following factors: agricultural and herbal planting development, building clay houses and dumping the building and garbage waste products near to residential areas which end to the attraction of sand fly vectors and rodents reservoirs close to the Shiraz city (7).

One of the interesting and challenging findings of the current study was molecular identification of *C. fasiculate* from seven samples of the CL patients. Studies have shown that *C. fasiculate*, as an insects parasite, is non-pathogenic to mammals (10); however, coinfection of *C. fasiculate* and *L. major* or *L. tropica* was reported in Khuzestan Province (southwest of Iran) (11). *Crithidia* in the lesion of right hand, *L. major* was isolated in left hand and *L. tropica* in face of CL patient (11). However, we report here in some patients only *Crithidia* without coinfection with other pathogenic *Leishmania* spp. It could be very important because lower trypanosomatid such as *Crithidia* in some situations such as immunosuppression caused diffuse cutaneous lesions (12). Moreover, *Crirhidia* could inter dermal mouse fibroblasts and stay inside these cells and could resist lysis by the complement system (12). All of the isolated samples were cultured and DNA was extracted from cultured samples, one of the possible hypothesis that why *Crithidia* was reported in the current study could be its ability to strongly develop and rapidly multiplicate inside culture media and destroy immediately other *Leishmania* parasites in coinfection situations. Therefore, when we isolated the parasite from active lesions both nonpathogenic *Crithidia* and pathogenic *Leishmania* spp. were present, but in culture, *Crithidia* was probably dominant parasite and killed other species.

Furthermore, the highest frequency was observed in 30–39 yr old, it can be caused by more outdoor activity of this group (1). The most affected part of the patient’s body was belonged to hand and face respectively, which has also been observed in the other distinct studies in various parts of the country (6, 7).

## Conclusion

Both *L. major* and *L. tropica* are circulating in Shiraz City as an endemic foci with high infection rates; however, *L. major* is predominant species of *Leishmania* in this region. Isolation of *Crithidia* in different sites of body can approve the existence of this nonpathogenic insect’s parasite in confirmed CL patients. Along with microscopic method, molecular and phylogenetically methods are necessary to determine the exact causative species of CL in endemic areas.

## Ethical considerations

Ethical issues (Including plagiarism, informed consent, misconduct, data fabrication and/or falsification, double publication and/or submission, redundancy, etc.) have been completely observed by the authors.
